# High-Throughput Virtual Screening, Molecular Dynamics Simulation, and Enzyme Kinetics Identified ZINC84525623 as a Potential Inhibitor of NDM-1

**DOI:** 10.3390/ijms20040819

**Published:** 2019-02-14

**Authors:** Md Tabish Rehman, Mohamed F AlAjmi, Afzal Hussain, Gulam Mohmad Rather, Meraj A Khan

**Affiliations:** 1 Department of Pharmacognosy, College of Pharmacy, King Saud University, Riyadh 11451, Saudi Arabia; malajmii@ksu.edu.sa (M.F.A.); afihussain@ksu.edu.sa (A.H.); 2 Rutgers Cancer Institute of New Jersey, Rutgers, The State University of New Jersey, New Brunswick, NJ 08901, USA; ratherbiotech@gmail.com; 3 Program in Translational Medicine, Peter Gilgan Centre for Research and Learning, The Hospital for Sick Children, Toronto, ON M5G 0A4, Canada

**Keywords:** antibiotic resistance, β-lactamases, *Escherichia coli*, *Klebsiella pneumoniae*, carbapenemase, molecular docking and simulation

## Abstract

The bacteria expressing New Delhi Metallo-β-lactamase-1 (NDM-1) can hydrolyze all β-lactam antibiotics including carbapenems, causing multi-drug resistance. The worldwide emergence and dissemination of gene *bla*_NDM-1_ (produces NDM-1) in hospital and community settings, rising problems for public health. Indeed, there is an urgent need for NDM-1 inhibitors to manage antibiotic resistance. Here, we have identified novel non-β-lactam ring-containing inhibitors of NDM-1 by applying a high-throughput virtual screening of lead-like subset of ZINC database. The screened compounds were followed for the molecular docking, the molecular dynamics simulation, and then enzyme kinetics assessment. The adopted screening procedure funnels out five novel inhibitors of NDM-1 including ZINC10936382, ZINC30479078, ZINC41493045, ZINC7424911, and ZINC84525623. The molecular mechanics-generalized born surface area and molecular dynamics (MD) simulation showed that ZINC84525623 formed the most stable complex with NDM-1. Furthermore, analyses of the binding pose after MD simulation revealed that ZINC84525623 formed two hydrogen bonds (electrostatic and hydrophobic interaction) with key amino acid residues of the NDM-1 active site. The docking binding free energy and docking binding constant for the ZINC84525623 and NDM-1 interaction were estimated to be −11.234 kcal/mol, and 1.74 × 10^8^ M^−1^ respectively. Steady-state enzyme kinetics in the presence of ZINC84525623 show the decreased catalytic efficiency (i.e., *k*_cat_/*K*_m_) of NDM-1 on various antibiotics. The findings of this study would be helpful in identifying novel inhibitors against other β-lactamases from a pool of large databases. Furthermore, the identified inhibitor (ZINC84525623) could be developed as efficient drug candidates.

## 1. Introduction

Globally, β-lactam antibiotics have been extensively used for the treatment of bacterial infections for decades owing to their high efficacy, affordability and low toxicity [1]. The overuse of β-lactam antibiotics resulted in the enormous rise of antibiotic resistance in bacteria. The worldwide spread of antibiotic resistance in clinical, as well as in community settings, has raised concern for the clinical use of β-lactam antibiotics [2]. Bacteria have the most prevalent mechanism of inactivating β-lactam antibiotics by expressing β-lactamases. This enzyme acts as a bacterial defense weapon to hydrolyze the β-lactam ring core of β-lactam antibiotics. Therefore, the antibiotics resistance necessitates the need for identifying novel inhibitors that lack the β-lactam ring [3]. The advantage of non-β-lactam inhibitors lies in the fact that such inhibitors cannot be easily hydrolyzed by β-lactamases and can escape the defense mechanisms of resistant bacteria [4]. 

According to Ambler’s molecular classification, β-lactamases are broadly classified into four classes as A, B, C and D [5]. The β-lactamases in classes A, C, and D, are serine β-lactamases as they employ an active site serine residue for the nucleophilic attack, while class B, β-lactamases use metal ions (zinc ions) during hydrolysis and, hence, are known as metallo-β-lactamases (MBLs). MBLs pose a great threat due to their broad-spectrum activities against the β-lactam core containing antibiotics. MBLs can hydrolyze virtually all the classes of β-lactam antibiotics except monobactams [1,2,3]. MBLs are further divided into the B1, B2, and B3 subgroups; with subgroup B1 MBLs having the most clinical relevance. The newly identified NDM-1 is a class B1 MBL expressed in pathogenic bacteria of clinical and community settings [5]. More recently, Bush et al. (1995, 2010) have classified β-lactamases on the basis of their functionality and divided them into three groups, namely, group 1 (cephalosporinases), group 2 (Oxacillinases, Penicillinases, extended-spectrum cephalosporinases, serine-based carbapenemases), and group 3 (metal-based carbapenemases) [6,7]. Thus, according to the classification of β-lactamases by Bush et al., NDM-1 belongs to group 3 [6,7]. Major Gram-negative pathogens such as *Escherichia coli*, *Enterobacter cloacae*, *Pseudomonas aeruginosa*, and *Acinetobacter baumanii* have been reported to produce MBLs, hence, making them a serious public health concern [8,9]. 

The widespread dissemination of NDM-1 has a significant impact on the treatment of hospital- and community-acquired infections [10]. The development of broad-spectrum MBL inhibitors is challenging because of (i) the structural variation across and within subclasses/ groups, and (ii) the requirement for selective inhibition of bacterial MBLs over human MBL-fold enzymes. The continued emergence of new MBL variants with altered substrate selectivity creates more challenges to the development of inhibitors [10]. Indeed, there is an urgent need for devising inhibitors to control the resistivity and increase the efficacies of antibiotics. 

In the present study, we employed Schrödinger’s small molecule suite (Schrödinger, LLC, New York, NY, USA) to identify novel non-β-lactam ring-containing inhibitors against NDM-1 by high throughput virtually screening (HTVS) of a lead-like subset of the ZINC database. Molecular docking was performed by both standard precision (SP) and extra precision (XP) docking methods. The compounds showing a good binding affinity (top 5%) were selected for further analysis. The physiological properties of the selected compounds were determined from the PubChem database, while the ADME/T (Adsorption, Distribution, Metabolism, Excretion, and Toxicity) properties were evaluated using QikProp (Schrödinger, LLC, New York, NY, USA). The effect of solvent on the stability of the protein-inhibitor complex was evaluated by MM-GBSA (Molecular Mechanics-General Born Surface Area) estimation. The compound with the lowest MM-GBSA value was finally subjected to molecular dynamics (MD) simulation to access the stability of the identified compound and NDM-1 complex. We have identified ZINC84525623 from the lead-like subset of the ZINC database as a potential non-β-lactam core containing novel inhibitors of NDM-1. Further, the potential of ZINC84525623 to inhibit NDM-1 was evaluated by performing steady-state enzyme kinetics against various antibiotics. To the best of our knowledge, this is the first study to report the inhibitory potential of ZINC84525623 against the NDM-1 enzyme.

## 2. Results and Discussion

Here we have applied various steps to screen, identify and validate potential NDM-1 inhibitors. The X-ray crystal structure of NDM-1 with hydrolyzed Meropenem at the active site (PDB Id: 4EYL) was used throughout this study.

### 2.1. Virtual Screening and Molecular Docking of ZINC Lead-Like Compounds

Computational approach comprising virtual screening, molecular docking, and molecular dynamics (MD) simulation is a widely used method for the exploration of novel inhibitors against a target protein [11,12]. In the present study, we have performed virtual screening of lead-like compounds from the ZINC database to identify novel inhibitors against NDM-1. The lead-like subset of the ZINC database contains 6,053,287 compounds. After the initial screening, according to Lipinski’s rule of five [13], a total of 1,000,143 compounds were funneled out for further analyses. These compounds were prepared for docking with the help of LIGPREP (LigPrep, Schrödinger, LLC, New York, NY, USA) and subjected to HTVS. A total of 10,000 compounds (~1%) were selected from the output of HTVS and subjected to SP docking. On the basis of the SP docking score, the top 1% of the compounds (~100 compounds) were used for XP docking (Table S1). The XP docking helped in removing the false positives and the scoring function was much more stringent than the HTVS and SP docking. By applying a docking score cutoff of ≥7.5 kcal/mol, we identified five compounds with the maximum scores (ZINC10936382, ZINC30479078, ZINC41493045, ZINC7424911, and ZINC84525623), as enlisted in Table 1. These compounds were used for further assessing the physiochemical and ADME/T properties.

### 2.2. Physiochemical and ADME/T Properties

The physiochemical properties of the identified compounds (ZINC10936382, ZINC30479078, ZINC41493045, ZINC7424911, and ZINC84525623) were determined from the PubChem database (https://pubchem.ncbi.nlm.nih.gov/), as enlisted in Table 2. The molecular weights of these compounds ranges between 339.358–416.452 g/mol, and each comprises of 1–2 H-bond donor groups, and 4–6 H-bond acceptor groups. Total polar surface area (Tpsa) of the identified compounds were within the 65.4–89.2 Å^2^ range, they had zero net charges, with 3–5 rotatable bonds (Table 2). 

For any compound to exhibit drug-like properties, it must exhibit ADME/T properties [14]. The ADME/T properties of the identified compounds were calculated using QIKPROP (QikProp, Schrödinger, LLC, New York, NY, USA) and are enlisted in Table 3. QIKPROP analysis comprises certain standard limits such as the values for polarizability (QPpolrz), aqueous solubility (QPlogS), hexadecane/gas (QPlogPC16), octanol/gas (QPlogPoct), water/gas (QPlogPw), octanol/water (QPlogPo/w), skin permeability (QPlogKp), and Khsa serum protein binding (QPlogKhsa) having standard ranges as 13 to 70, −6 to 0.5, 4 to 18, 8 to 43, 5 to 48, −2 to 6, −8.0 to −1.0, and 1.5 to 1.2, respectively [14]. The analysis of ADME/T properties shows values of QPpolrz, QPlogS, QPlogPC16, QPlogPoct, QPlogPw, QPlogPo/w, QPlogKp, and QPlog Khsa in the permitted range (Table 3). Based on the collective data, we infer that the ADME/T properties of ZINC10936382, ZINC30479078, ZINC41493045, ZINC7424911, and ZINC84525623 are within the prescribed limits for a potential candidate drug compound.

### 2.3. Analyses of the NDM-1 and Screened Inhibitors Interaction

#### 2.3.1. NDM-1 and Meropenem Interaction

**Figure 1:**
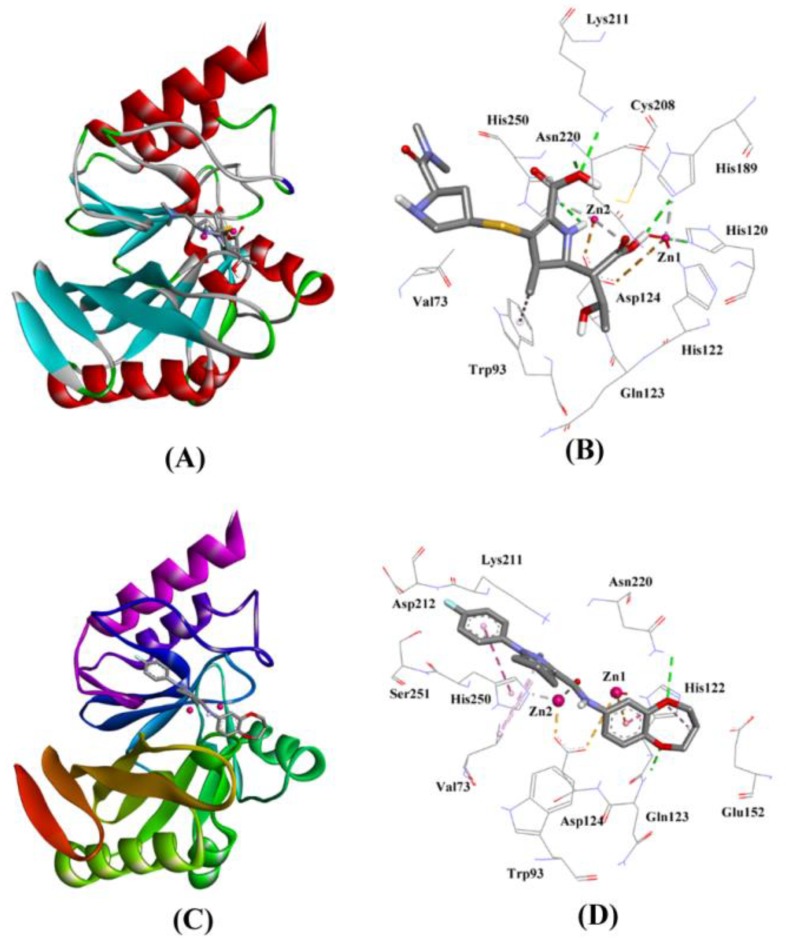
The molecular docking of hydrolyzed Meropenem and ZINC10936382 with NDM-1. (**A**) The binding of Meropenem at the active site pocket of NDM-1, (**B**) Molecular interaction between the amino acid residues of NDM-1 and hydrolyzed Meropenem, (**C**) Binding of ZINC10936382 at the active site of NDM-1, (**D**) Molecular interaction between the amino acid residues of NDM-1 and ZINC10936382.

The molecular docking of Meropenem at the active site of NDM-1 revealed that it fits into the active site cavity (Figure 1A,B). The complex was stabilized by six hydrogen bonds (with His120, His189, Lys211, Asn220, His250) and one hydrophobic interaction with Trp93. Additionally, some other residues such as Val73, Trp93, His122, Glu123, Asp124, and Cys208 also provide stability to NDM-1 and Meropenem complex (Figure 1A,B). Further, Zn1 also participated in the interaction between Meropenem and NDM-1 by forming one metal-coordination bond. The docking binding energy (∆*G*) of the interaction and the corresponding docking binding affinity (*K*_d_) were estimated to be −6.413 kcal/mol and 5.05 × 10^4^ respectively (Table 4).

#### 2.3.2. NDM-1 and ZINC10936382 Interaction

The interaction between ZINC10936382 and NDM-1 was predicted by performing molecular docking. We found that ZINC10936382 binds at the active site of NDM-1 (Figure 1C,D) by forming two hydrogen bonds with Gln123 and Asn220, and five hydrophobic interactions with Val73, His122 and His250. Moreover, Zn1 and Zn2 also formed one electrostatic and one metal-coordination bond with ZINC10936382. Some charged and polar residues such as Trp93, Asp124, Glu152, Lys211, Asp212, and Ser251 were involved in stabilizing the ZINC10936382-NDM complex. The docking binding energy (∆*G*) of stabilization was found to be −8.322 kcal/mol, corresponding to a docking binding affinity (*K*_d_) of 1.27 × 10^6^ M^−1^ (Table 4). Based on the data, it is evident that ZINC10936382 interacted with the key residues of NDM-1 which participate in making contacts with the β-lactam substrate.

#### 2.3.3. NDM-1 and ZINC30479078 Interaction

**Figure 2:**
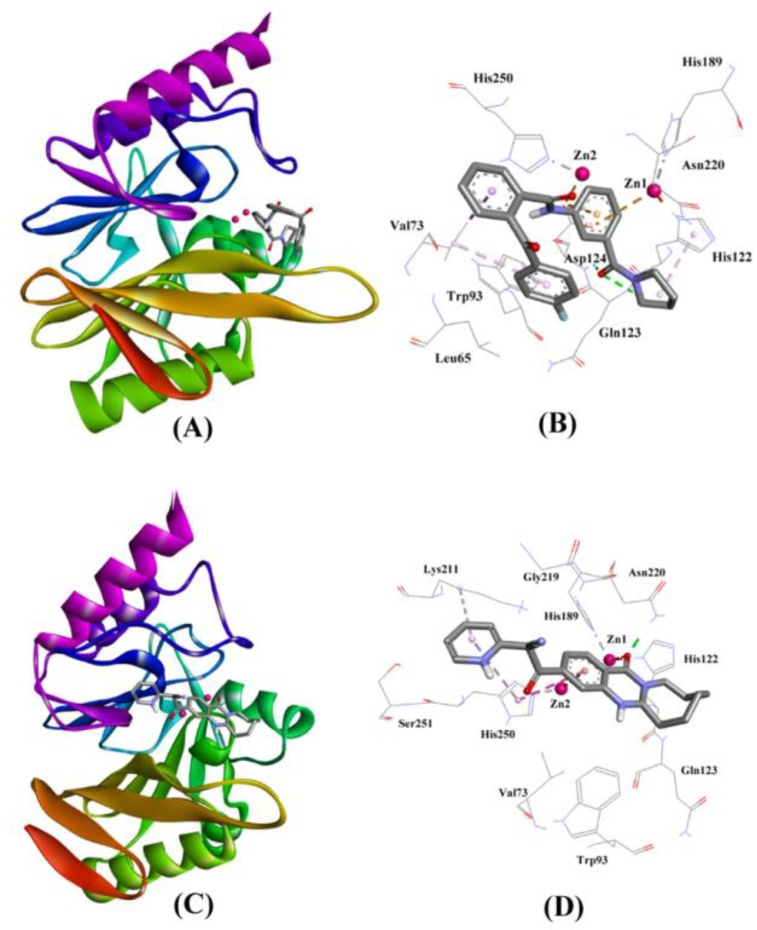
The molecular docking of ZINC30479078, and ZINC41493045 with of NDM-1. (**A**) The binding of ZINC30479078 at the active site cavity of NDM-1, (**B**) The molecular interactions between the amino acid residues of NDM-1 and ZINC30479078, (**C**) The binding of ZINC41493045 at the active site cavity of NDM-1, and (**D**) the molecular interactions between the amino acid residues of NDM-1 and ZINC41493045.

The molecular docking of ZINC30479078 with NDM-1 revealed that it fits into the active site and was stabilized by two hydrogen bonds with Gln123 (2.67 Å) and Asp124 (2.18 Å), and one electrostatic interaction with Asp124 (3.70 Å). Moreover, the ZINC30479078-NDM-1 complex was stabilized by two hydrophobic interactions with Val73 and one with His122 (Figure 2A,B and Table 4). Other amino acid residues which participate in the interaction were Leu65, Trp93, His189, Asn220, and His250. The docking binding energy (∆*G*) of stabilization for the NDM-1-ZINC30479078 complex was estimated to be −9.046 kcal/mol, corresponding to a docking binding affinity (*K*_d_) of 4.31 × 10^6^ M^−1^ (Table 4).

#### 2.3.4. NDM-1 and ZINC41493045 Interaction

The affinity of ZINC41493045 towards the active site of NDM-1 was predicted by performing molecular docking in the extra precision (XP) mode in MAESTRO (Maestro, Schrödinger, LLC, New York, NY, USA). Figure 2C clearly shows that ZINC41493045 was comfortably fitted at the active site cavity of NDM-1. It interacted with His122 and Asn220 through hydrogen bonding, while Lys211 and His250 were involved in hydrophobic interactions (Figure 2D). Moreover, Zn1 formed one metal-coordination bond while Zn2 formed one electrostatic interaction with ZINC41493045. The complex was stabilized by a docking binding energy (∆*G*) of −7.714 kcal/mol, which corresponded to a docking binding affinity (*K*_d_) of 4.54 × 10^5^ M^−1^ (Table 4).

#### 2.3.5. NDM-1 and ZINC7424911 Interaction

**Figure 3:**
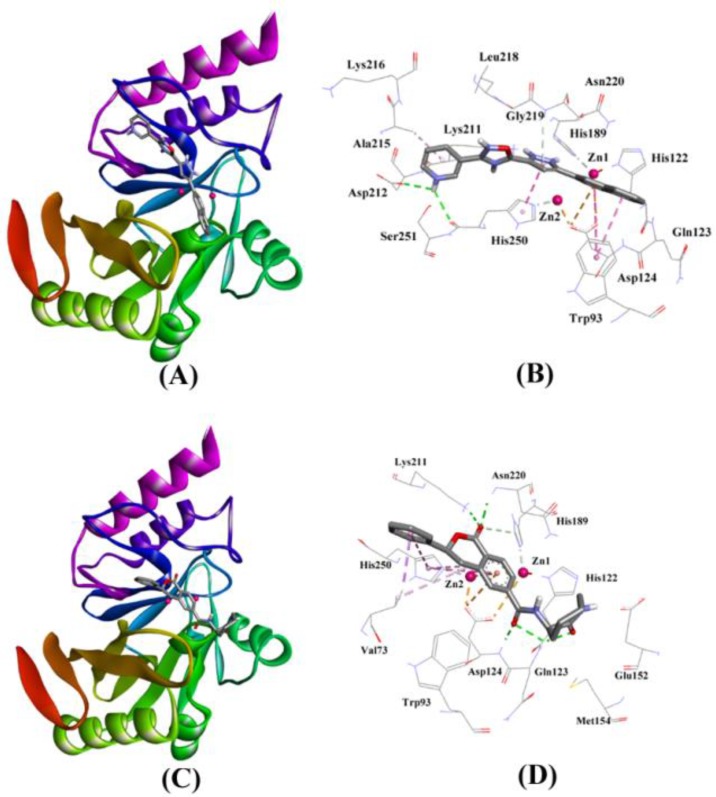
The molecular docking of ZINC7424911, and ZINC84525623 with of NDM-1. (**A**) The binding of ZINC7424911 at the active site cavity of NDM-1, (**B**) the molecular interactions between the amino acid residues of NDM-1 and ZINC7424911, (**C**) the binding of ZINC84525623 at the active site cavity of NDM-1, and (**D**) the molecular interactions between the amino acid residues of NDM-1 and ZINC84525623.

The molecular docking of ZINC7424911 with NDM-1 revealed that it was bound deep into the active site cavity of NDM-1 (Figure 3A,B). The NDM-1-ZINC7424911 complex was stabilized by three hydrogen bonds with Asp212, Asn220, and His250; four hydrophobic interactions with Trp93, Ala215 and His250; and one electrostatic interaction with Asp124. Moreover, Gly219 along with some polar and charged residues like His122, Gln123, His189, Lys211, Lys216, Leu218, and Ser251 also participated in stabilizing the NDM-1-ZINC7424911 complex (Figure 3B). The docking binding energy (∆*G*) and docking binding affinity (*K*_d_) of ZINC7424911 towards NDM-1 were estimated to be −8.254 kcal/mol and 1.13 × 10^6^ M^−1^, respectively (Table 4).

#### 2.3.6. NDM-1 and ZINC84525623 Interaction

The molecular docking revealed that ZINC84525623 binds at the active site of the enzyme (Figure 3C,D). The ZINC84525623-NDM-1 complex was stabilized by five hydrogen bonds with Gln123 (2.16 Å and 2.66 Å), Asp124 (2.08 Å), Lys211 (2.71 Å), and Asn220 (2.10 Å); one carbon-hydrogen bond with His189 (3.57 Å), and two electrostatic interactions with Zn2 (3.66 Å) and Asp124 (4.33 Å). Further, ZINC84525623 formed two hydrophobic interactions with Val73 (Pi–Sigma, and Alkyl), and three hydrophobic interactions with His250 (two Pi–Pi, and one Pi–Alkyl). Additionally, ZINC84525623 was surrounded by some other residues such as Trp93, His122, Glu152, and Met154 (Figure 3D). The docking binding energy (∆*G*) and docking binding affinity (*K*_d_) for ZINC84525623 and NDM-1 interaction were estimated to be −8.790 kcal/mol and 2.80 × 10^6^ M^−1^, respectively (Table 4). 

The X-ray crystal structure of NDM-1 is characterized by a four-layered αβ/βα fold with a deep and wide catalytically active site [15,16]. The active site of NDM-1 harbors two Zinc ions; Zn1 binds His120, His122, and His189 arranged in a tetrahedral geometry, while Zn2 adopts a trigonal pyramidal geometry surrounded by Asp124, Cys208, and His250 [17]. A water molecule located between Zn1 and Zn2 acts as a nucleophile during hydrolysis of the β-lactam ring of antibiotics. An insight into the mechanism of hydrolysis by NDM-1 suggests the significant role played by the above-mentioned residues in maintaining the overall structure and function of NDM-1. Our results from this study indicate that the shortlisted compounds (ZINC10936382, ZINC30479078, ZINC41493045, ZINC7424911, and ZINC84525623) from the ZINC database interacted with the key active site residues of NDM-1 such as His122, Asp124, His189, Cys208, His250, Zn1, and Zn2. Moreover, hydrophobic residues around the active site also play a significant role in recognizing the substrate and presenting them to the active site in the proper orientation [18]. For instance, the binding of the substrate at the active site leads to the re-orientation of Met67 away from the di-zinc center by ~4.9 Å while Leu65 moves ~2.1 Å closer to the di-zinc center. This movement helps the R1 group of the substrate to interact with the hydrophobic patch made up of Leu65, Met67, and Trp93 and get accommodated into the active site of NDM-1 [18]. Moreover, Trp93 has been shown to provide conformational stability to the overall structure of NDM-1 [19]. We have found that the shortlisted compounds from the ZINC database (ZINC10936382, ZINC30479078, ZINC41493045, ZINC7424911, and ZINC84525623) were able to form stable interactions with the hydrophobic residues such as Leu65, Val73, Trp93, and Met154. Further, upon substrate binding, Asn220 is pulled ~1 Å closer to the di-zinc center, thus allowing its interaction with the β-lactam carbonyl group. Together with Zn1, Asn220 provides an oxy-anion hole to the active site, which polarizes the β-lactam carbonyl group and facilitates the nucleophilic attack by the hydroxide ion [20,21]. It has been suggested that the hydroxide ion is generated from water molecules attached to Asp124. Furthermore, Zn2 interacts with the amide nitrogen of the substrate and stabilizes the charge on it upon the hydrolysis of the peptide bond [22,23]. Our results also indicate that Asn220 was engaged in forming hydrogen bonds with ZINC10936382, ZINC41493045, ZINC07424911, and ZINC84525623. On the other hand, Zn2 interacted with ZINC41493045 and ZINC84525623 through electrostatic interactions while it formed a metal-acceptor (or metal-coordination) bond with ZINC10936382. Together, these results suggest that the selected compounds from the ZINC database have the potential to bind tightly at the active site of NDM-1 and, thus, could serve as potent inhibitors. 

### 2.4. Molecular Mechanics—General Born Surface Area (MM-GBSA) Estimation

The effect of the solvent on the interaction between ZINC compounds and NDM-1 was evaluated by determining the MM-GBSA of the top 5% shortlisted compounds which have a ≥7.5 kcal/mol XP docking score (total 5 compounds). We found that the MM-GBSA of compounds ZINC10936382, ZINC30479078, ZINC41493045, ZINC7424911, and ZINC84525623 were −61.432, −70.643, −62.523, −60.619, and −96.388 kcal/mol, respectively (Table 4). The MM-GBSA of Meropenem (as control) has been found to be −52.971 kcal/mol. ZINC84525623 had the lowest value of MM-GBSA (−96.338 kcal/mol) amongst the screened compounds and it was also much lower than the Meropenem control (−52.971 kcal/mol). Altogether, the MM-GBSA results suggest the formation of a stable complex between NDM-1 and ZINC84525623. Further, the stability of the NDM-1-ZINC84525623 complex was estimated by performing MD simulation.

#### Molecular Dynamics (MD) Simulation

**Figure 4:**
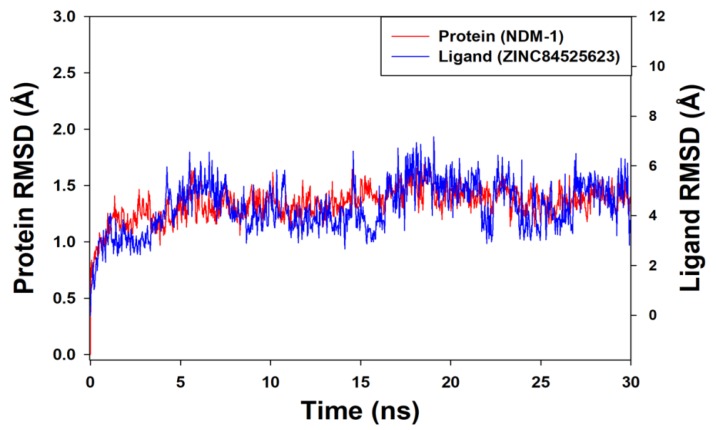
The molecular dynamic (MD) simulation of ZINC84525623 with NDM-1 depicting the root mean square deviations (RMSDs) of NDM-1 alone and the NDM-1-ZINC84525623 complex.

MD simulation is a commonly used computational estimation of the dynamics and stability of a receptor-ligand complex under physiological conditions. We have performed MD simulation on the initial conformation of the ZINC84525623-NDM-1 complex obtained after XP docking for 30 ns. The initial pose and the final pose of ZINC84525623 after MD simulation is shown in Figure S1. The results representing the RMSD of the protein backbone between the initial conformation and after the completion of MD simulation are shown in Figure 4. 

It is evident from Figure 4 that the protein backbone showed a large deviation from 0.8 Å to 1.2 Å during the first 5 ns, which is due to the stabilization of the protein’s initial structure. Thereafter, the system was stabilized and showed steady-state dynamics. The RMSD of the protein backbone in 5–30 ns fluctuated within 1.0–1.8 Å, which was, in fact, within the prescribed upper limit of 2.0 Å. Similarly, the RMSD of ZINC84525623 that fit on protein backbone was stabilized after 3 ns, thereafter it showed variation between 4.0–6.0 Å. The large variation in the RMSD of ZINC84525623 that fit on the NDM-1 backbone might be due to the entry of this big ligand into the active site cavity of NDM-1. Altogether, these results suggest the formation of a stable complex between ZINC84525623 and NDM-1 upon the formation of favorable interactions with key amino acid residues.

**Figure 5:**
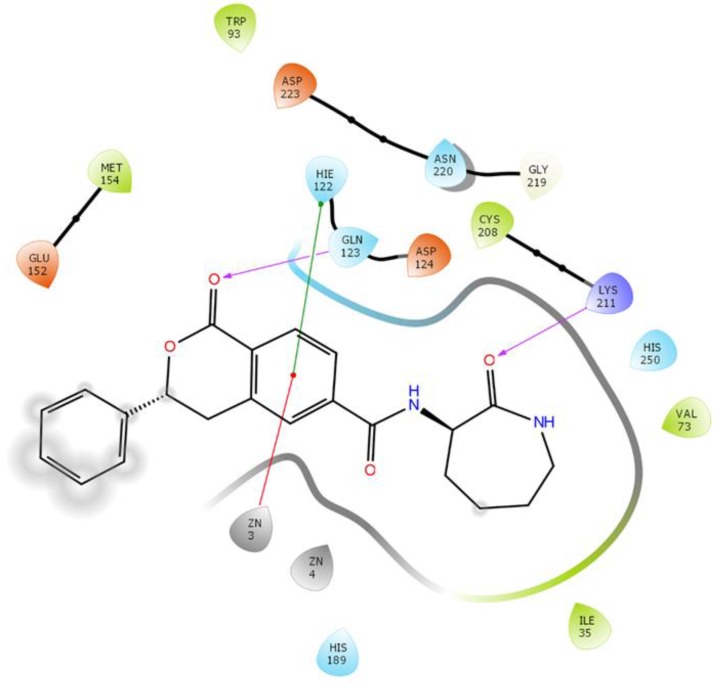
The interaction between ZINC84525623 and NDM-1 after molecular dynamic (MD) simulation. ZN3 and ZN4 correspond to Zn1 and Zn2 of the X-ray crystal structure. Hydrogen bonds, hydrophobic interaction, and electrostatic interaction are shown in pink, green, and red, respectively. Residues shown in red, green, blue, and purple are charged, hydrophobic, and polar residues, respectively. Glycine and metal ions are shown in white and black, respectively.

The analysis of the interaction between ZINC86525623 and NDM-1 after MD simulation revealed that it binds at the active site of the enzyme. The ZINC86525623-NDM-1 complex was stabilized by two hydrogen bonds with Gln123 and Lys211 along with one hydrophobic interaction with His122 and an electrostatic interaction with Zn1 (Figure 5). Other amino acid residues that surround the inhibitor were Ile35, Val73, Trp93, Asp124, Glu152, Met154, His189, Cys208, Gly219, Asn220, Asp223, His250, and Zn2. It should be noted that ZINC86525623 interacted with key active site residues such as His122, Asp124, His189, Cys208, His250, Zn1, and Zn2. The docking binding energy after MD simulation was found to be −11.234 kcal/mol, which corresponded to a binding affinity of 1.74 × 10^8^ M^−1^.

### 2.5. IC_50_ Value Determination

**Figure 6:**
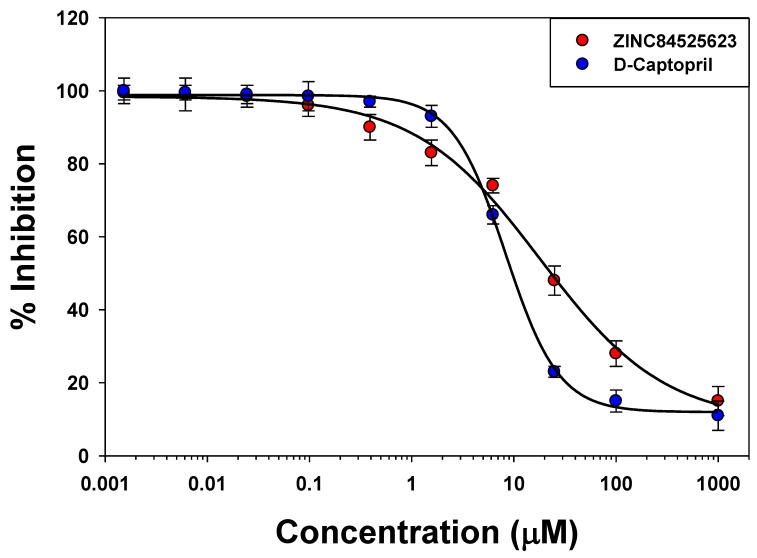
The determination of the IC_50_ values of ZINC84525623 (red), and d-captopril (blue).

To assess the inhibitory potency of the identified compound, IC_50_ values were calculated. Here, we have calculated the IC_50_ values of ZINC84525623 and D-captopril (+ve control; known NDM-1 inhibitor) (Figure 6) for assessing the inhibitory role of ZINC84525623. The % inhibition kinetics plot shows the IC_50_ values of ZINC84525623 and d-captopril as 18.2 and 8.3 µM, respectively. Previous study has also reported the IC_50_ value of d-captopril as ~7.9 µM [24]. Since the IC_50_ value of ZINC84525623 is only around 2-folds lower than that of d-captopril, this suggests the comparable inhibitory potential of ZINC84525623 to a known inhibitor, d-captopril. 

### 2.6. Steady State Enzyme Kinetics

Here we asked to see the effect of an identified inhibitor over the enzyme kinetics of NDM-1 against different antibiotics. The enzyme kinetics profiles of NDM-1 with various substrates in the presence or absence of ZINC84525623 have been shown in Figure S2. The kinetic parameters of the purified NDM-1 revealed that NDM-1 in the absence of the ZINC84525623 inhibitor can hydrolyze different β-lactam antibiotics including ampicillin, cefotaxime, imipenem, meropenem, as well as nitrocefin (a chromogenic substrate) (Figure S2, Table 5). NDM-1 displayed high affinity (*K*_m_ = 29.0–88.9 µM) and catalytic prowess (*k*_cat_/*K*_m_ = 4.93–9.73 µM^−1^ s^−1^) for different studied antibiotics. However, in the presence of ZINC84525623, the *K*_m_ values of different substrates increased up to 1.25–1.89 folds, while the *k*_cat_ values decreased by approximately 2.26–5.41 folds. The catalytic efficiency (*k*_cat_/*K*_m_) of NDM-1 in the presence of ZINC84525623 was reduced by approximately 3.13–7.37 folds (Table 5). The molecular basis of the decrease in the catalytic efficiency (*k*_cat_/*K*_m_) of NDM-1 in the presence of ZINC84525623 can be assessed by analyzing the binding pose of ZINC84525623 at the active site of NDM-1. As we discussed above, ZINC84525623 shows the binding at the key active site and with surrounding residues such as Ile35, Val73, Trp93, His122, Gln123, Asp124, Glu152, Met154, His189, Cys208, Lys211, Gly219, Asn220, Asp223, and His250 (Figure 5). Moreover, ZINC84525623 also formed an electrostatic interaction with the Zn1 ion. Collectively, ZINC84525623 formed a stable complex with NDM-1, making it unavailable for hydrolysis by antibiotics.

## 3. Materials and Methods

### 3.1. Materials

Ampicillin, cefotaxime, imipenem, meropenem, d-captopril, and PAR (i.e., 4-(2-Pyridylazo) resorcinol) were purchased from Sigma-Aldrich (St. Louis, MO, USA). Nitrocefin was purchased from Calbiochem (St. Louis, MO, USA). Other reagents and chemicals used in the present study were of analytical grade. The inhibitor ZINC84525623 was procured from Mcule Inc (Palo Alto, CA, USA).

### 3.2. Experimental Procedures

**Figure 7:**
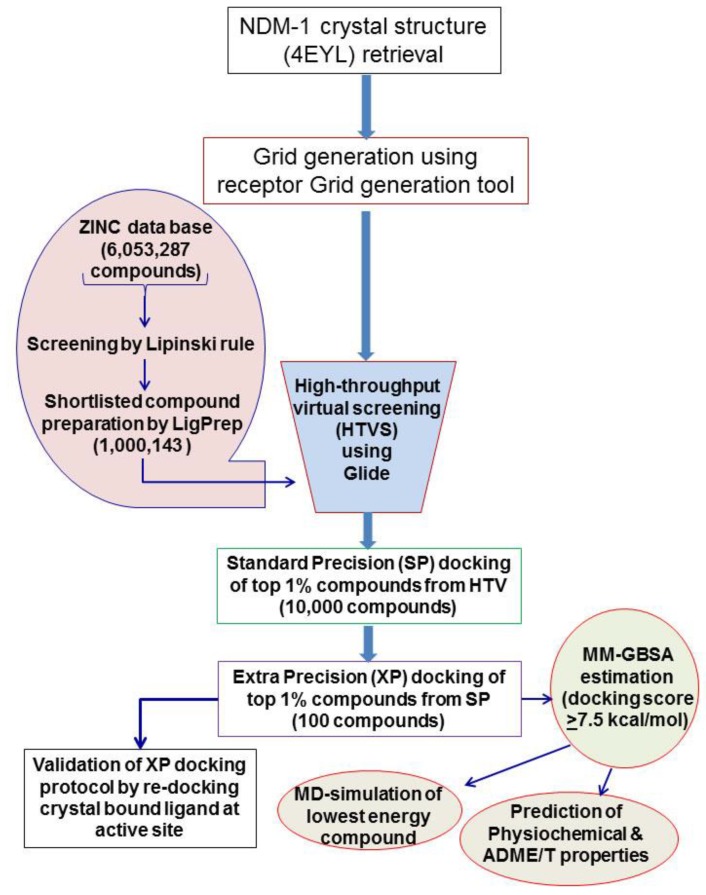
The flowchart of the various steps undertaken in the study to identify the novel inhibitor.

All the virtual screening and docking work was performed as described earlier [11,12]. Briefly, the docking steps were executed on a workstation with the following specifications: Windows 7, Intel^®^ Xenon^®^ E3-1245v5 8C, 3.50 GHz, 28.0 GB RAM, 1 TB hard disc, and an NVIDIA Quadro M2000 graphics card. Furthermore, molecular dynamics (MD) simulations were conducted on a Linux-based workstation equipped with an NVIDIA Tesla K20 GPU card. MAESTRO (Maestro version 11.5.011, Schrödinger, LLC, New York, NY, USA) was used for all the steps involved in the protein and ligand preparation, receptor grid generation and docking. MD simulation was performed using DESMOND (Desmond version 11.5.011, Schrödinger, LLC, New York, NY, USA). The various steps, algorithmic procedures and methodologies such as virtual screening, molecular docking, and molecular dynamics simulations are summarized in a schematic flowchart (Figure 7).

### 3.3. Retrieval and Preparation of Ligands

The lead-like subset of the ZINC database (http://zinc.docking.org/) was screened to identify novel non-beta-lactam ring containing inhibitors of NDM-1 using GLIDE (Glide, Schrödinger, LLC, New York, NY, USA). The lead-like subset of the ZINC database contains 6,053,287 compounds in the ready-to-dock format (accessed on 5 September 2017). These compounds were shortlisted into 1,000,143 compounds by applying Lipinski’s rule of five [13] and prepared in LIGPREP (LigPrep, Schrödinger, LLC, New York, NY, USA). The ionization states of compounds were generated at pH 7.0 ± 2.0 with the help of EPIK (Epik, Schrödinger, LLC, New York, NY, USA) followed by the removal of salts from ligands. A maximum of 32 stereoisomers was generated for a compound. An OPLS3 (Optimized Potentials for Liquid Simulations) force field was used to minimize the energy of each compound with default parameters.

### 3.4. Preparation of Protein, Active Site Prediction, and Grid Generation

The X-ray crystal structure of NDM-1 in a complex with hydrolyzed Meropenem (PDB Id: 4EYL) at a resolution of 1.90 Å was retrieved from the PDB database (http://www.rcsb.org/pdb) [15]. The structure of NDM-1 was prepared using the protein preparation wizard of GLIDE (Glide, Schrödinger, LLC, New York, NY, USA) as described earlier [25]. Briefly, the missing hydrogen atoms were added, assigned bond orders, created zero bond order for disulfide bonds, and any other hetero-atoms present in the structure were deleted. The proper charge of the Zinc ions was assigned. Crystallographic water molecules were removed while the catalytic water molecule was kept intact. PRIME (Prime, Schrödinger, LLC, New York, NY, USA) was used to add missing loops and any side chains of the residues. The H-bond network was created before energy minimization using the OPLS3 force field with a default constraint of 0.30 Å RMSD (Root Mean Square Deviation) [26]. The grid box was generated by selecting the center of the bound ligand (i.e., Meropenem) as the centroid of the grid box (30 Å × 30 Å × 30 Å) in the receptor-grid generation module of MAESTRO (Maestro version 11.5.011, Schrödinger, LLC, New York, NY, USA).

### 3.5. Virtual Screening and Molecular Docking

The molecular docking of lead-like compounds from the ZINC database (filtered by Lipinski’s rule of five) was performed using GLIDE (Glide, Schrödinger, LLC, New York, NY, USA). All the shortlisted compounds were docked at the active site of NDM-1 using the HTVS (High Throughput Virtual Screening) scoring function to estimate protein-ligand binding affinities. Standard precision (SP) docking was performed on the compounds filtered through HTVS. The protein-compound complex was optimized by post-docking minimization. Extra precision (XP) docking was performed on the compounds shortlisted after SP docking by keeping the parameters of scaling factor and partial charge cutoff at 0.80 and 0.15, respectively. Post-docking analysis and visualization were performed on MAESTRO (Maestro, Schrödinger, LLC, New York, NY, USA). The docking binding affinity of compounds for the NDM-1 active site was calculated from the docking binding energy as described earlier using the following relation [27].

(1) $$\Delta G=-RT\ln K_{d}$$

where Δ*G* is the change in docking binding energy, *T* is the temperature, R is the Boltzmann gas constant (R = 1.987 cal/mol/K), and *K*_d_ is the docking binding affinity.

### 3.6. Validation of Docking Protocol

The validity of the docking protocol was confirmed by performing XP docking of the X-ray crystal structure ligand (i.e., Meropenem) at the active site of NDM-1 and comparing the RMSDs between the docked pose and the crystal structure pose of Meropenem, as shown in the supplementary figure (Figure S3). We found that meropenem occupied a similar position at the active site of NDM-1. 

### 3.7. Determination of Physiochemical and ADME/T Properties

The physiochemical properties of the top 5% compounds in XP docking were determined from the PubChem database (https://pubchem.ncbi.nlm.nih.gov/). The drug-likeliness of the selected compounds from the ZINC database was evaluated by determining the absorption, distribution, metabolism, excretion and toxicological (ADME/T) properties using QIKPROP (QikProp, Schrödinger, LLC, New York, NY, USA). 

### 3.8. Molecular Mechanics—Generalized Born Surface Area (MM-GBSA) Calculations

The effect of the solvent on the binding free energies of the top 5% compounds in XP docking was estimated using molecular mechanics force fields and implicit solvation using the MM-GBSA method of PRIME (Prime, Schrödinger, LLC, New York, NY, USA). The pose viewer file was used to generate binding energy calculations. The docked poses were minimized using the local optimization feature in PRIME (Prime, Schrödinger, LLC, New York, NY, USA) and binding free energies of shortlisted compounds were computed using the MM-GBSA continuum solvent model which incorporates the OPLS3 force field [26], the VSGB solvent model [28], and rotamer search algorithms [29]. The binding energy was calculated based on the following equation.

(2) $$\Delta G=E_{Complex(Minimized)}-\left[E_{Ligand(Minimized)}+E_{Receptor(Minimized)}\right]$$

For MM-GBSA calculations, all the protein atoms were kept rigid while relaxing the atoms of the compounds. Furthermore, binding free energy calculations were used to rank the protein-compound complexes.

### 3.9. Molecular Dynamics (MD) Simulation

The stability of the docked complex was determined by performing an MD simulation as described previously [11,25]. The protein-ZINC compound complex with the minimum MM-GBSA binding energy was selected for the 30 ns MD simulation using DESMOND (Desmond, Schrödinger, LLC, New York, NY, USA). The system builder panel was used to prepare an orthorhombic simulation box with the TIP3P explicit water model. A minimum distance of 10 Å between the protein surface and the boundary of the simulation box was maintained. A total of 21 Na^+^ and 20 Cl^−^ counterions were added to neutralize the system and the isosmotic salt environment was maintained by adding 150 mM NaCl. The non-bonded model was used for the Zn ions. The system was minimized with 2000 iterations with a convergence criterion of 1 kcal/mol/Å. The minimized system was subjected to a 30 ns MD simulation using the NPT (normal Pressure and Temperature) ensemble at 300 K and 1.013 bars with the default setting of relaxation before simulation. The Nose-Hoover Chain thermostat [30] and Martyna–Tobias–Klein barostat [31] were used to maintain the temperature and pressure, respectively. A time step of 2 fs was considered during the simulation and, at every 10 ps, the energy and structure were recorded and saved in the trajectory. Three-dimensional structures and trajectories were visually inspected using MAESTRO (Maestro, Schrödinger, LLC, New York, NY, USA).

### 3.10. Cloning, Expression and Purification of NDM-1

The protein expression and purification service of GenScript (New Jersey, NJ, USA) were used for the cloning, expression and purification of NDM-1 (Figure S4). Briefly, the target sequence of NDM-1 without signal peptide was optimized for codon bias and synthesized. The synthesized sequence was then cloned into the pET30a vector with His-tag at the N-terminal end. The recombinant plasmid was used to transform *E. coli* BL21 Star (DE3) cells. A single colony was inoculated into the LB medium containing kanamycin and the culture was incubated at 37 °C with 200 rpm shaking. The culture was induced with IPTG and the expression of the protein was monitored for different time intervals using SDS-PAGE. The expression of NDM-1 was scaled-up by inoculating BL21 Star (DE3) cells in TB medium containing kanamycin at 37 °C. When the OD_600_ reached 1.0–1.2, the culture was induced with IPTG at 15 °C for 16 h and then centrifuged to harvest the cells. Cell pellets were resuspended in a lysis buffer followed by sonication. After centrifugation, the supernatant was further purified using a Ni-NTA column. The purified protein was dialyzed against a 50 mM HEPES buffer (pH 7.0) containing 250 mM NaCl and 100 µM ZnCl_2_. The molecular weight and purity were confirmed by SDS-PAGE and Western blotting (Figure S5). The concentration of the protein was determined spectrophotometrically using a molar extinction coefficient of 27,800 M^−1^ cm^−1^. A PAR (4-(2-Pyridylazo) resorcinol) assay was used to determine the zinc content of the purified NDM-1 protein. We found that NDM-1 contained 1.89 ± 0.4 equivalent Zn(II) ions for each NDM-1 molecule.

### 3.11. Determination of IC_50_

The IC_50_ values of ZINC and d-captopril (known inhibitor) were determined after incubating various concentrations (0.001 to 1000 µM range) of inhibitors with the purified NDM-1 protein for 5 min at 30 °C followed by monitoring the hydrolysis of 100 µM nitrocefin [32]. The IC_50_ value is defined as the concentration of the inhibitor which inhibited the enzyme activity by 50%.

### 3.12. Enzyme Kinetics

The effect of the shortlisted inhibitor on the activity of NDM-1 was determined by performing steady-state enzyme kinetics as described previously [26]. Briefly, the hydrolysis of the following antibiotics was monitored in the absence and presence of the inhibitor: nitrocefin (∆ε_486_ = +15,000 M^−1^ cm^−1^), ampicillin (∆ε_235_ = −900 M^−1^ cm^−1^), cefotaxime (∆ε_264_ = −7250 M^−1^ cm^−1^), imipenem (∆ε_295_ = −10,500 M^−1^ cm^−1^), and meropenem (∆ε_297_ = −10,940 M^−1^ cm^−1^). All the reactions were performed in a 50 mM HEPES buffer pH 7.0 supplemented with 250 mM NaCl and 100 µM ZnCl_2_ at 30 °C as reported earlier [33]. To the buffer, 20 µg/mL BSA was added to avoid the denaturation of NDM-1. It has been found that BSA did not show any effect on the hydrolytic ability of NDM-1. Kinetic parameters (*k*_cat_ and *K*_m_) were deduced by converting the observed hydrolysis into initial velocities and fitting them into the following Michaelis–Menten equation.

(3) $$v=\frac{V_{\max}[S]}{K_{m}+[S]}$$

(4) $$k_{cat}=\frac{V_{\max}}{\left[E\right]}$$

where, *v* and *V*_max_ are the initial and maximum velocities respectively. [S] and [E] are the molar concentrations of substrate and enzyme, respectively.

## 4. Conclusions

The dissemination of NDM-1 in bacteria has led to the emergence of resistance of bacteria towards commonly used antibiotics. This scenario has pushed us back into the pre-antibiotic era, whereby treating simple bacterial infections is life-threatening. Here, we have attempted to identify novel non-β-lactam ring-containing inhibitor against NDM-1. We employed high throughput virtual screening to screen the ZINC database of lead-like compounds (6,053,287 compounds). These compounds were filtered into 1,000,143 compounds according to Lipinski’s rule of five and then subjected to HTVS followed by SP docking on 10,000 compounds (top 1%), and XP docking on 100 compounds (top 1%) using GLIDE (Glide, Schrödinger, LLC, NY, USA). After molecular docking, we have identified 5 compounds (top 5%), namely, ZINC10936382, ZINC30479078, ZINC41493045, ZINC7424911, and ZINC84525623 as the potential inhibitors of NDM-1. On further investigation through MM-GBSA calculations on the above-selected compounds, we have identified that ZINC84525623 formed the most stable complex with NDM-1. Insight into the molecular interaction between ZINC84525623 and NDM-1 was gained by molecular docking, and the stability of the NDM-1-ZINC84525623 complex was assessed by a molecular dynamics simulation. Altogether, findings indicate that ZINC84525623 has a strong interaction with the key active site residues of NDM-1 through hydrogen bonds, electrostatic and hydrophobic interactions. The potential of ZINC84525623 to inhibit the purified NDM-1 enzyme was evaluated by monitoring the steady-state enzyme kinetic on various antibiotics. The NDM-1 enzyme showed increased *K*_m_ values along with reduced *k*_cat_ values leading to substantial decreased *k*_cat_/*K*_m_ values. To the best of our knowledge, this is the first study wherein ZINC84525623 has been reported to be a potent inhibitor of NDM-1. Since ZINC84525623 is a non-β-lactam ring-containing compound, the resistant bacteria will have to adapt themselves to hydrolyze it. Moreover, bacteria will need time to evolve in order to develop new resistance mechanisms against ZINC84525623. We believe this research will give a new dimension into the discovery of a non-β-lactam core containing inhibitors against metallo-β-lactamases.

## Figures and Tables

**Table 1:** The extra precision (XP) docking parameters of the identified compounds by high precision virtually screening (HTVS) and standard precision (SP) docking.

S. No.	ZINC ID	Docking Score *	Glide g-Score *	Glide e-Model *	XP g-Score *
1.	ZINC10936382	−8.322	−8.322	−68.183	−8.322
2.	ZINC30479078	−9.046	−9.046	−66.578	−9.046
3.	ZINC41493045	−7.714	−7.714	−64.597	−7.714
4.	ZINC7424911	−8.254	−8.265	−63.254	−8.254
5.	ZINC84525623	−8.790	−8.790	−64.740	−8.790
6.	Control (Meropenem)	−6.413	−6.413	−56.140	−6.413

* All the energies are in kcal/mol. The compounds shown in bold were shortlisted for further analysis. HTVS, SP, and XP stand for High Throughput Virtual Screening, Standard Precision, and Extra Precision respectively.

**Table 2:** The physiochemical properties (PubChem database) of the top 5% selected compound through XP ^a^ docking scores.

Structure/Name of Compounds	Mol. Weight (g/mol)	HBD ^b^	HBA ^c^	Tpsa ^d^ (Å^2^)	Net Charge	RB ^e^
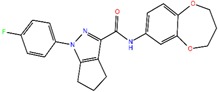 ZINC10936382	393.418	1	5	65.4	0	3
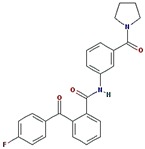 ZINC30479078	416.452	1	4	66.5	0	5
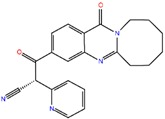 ZINC41493045	372.428	0	6	89.2	0	3
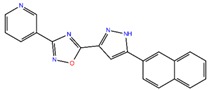 ZINC7424911	339.358	1	5	80.5	0	3
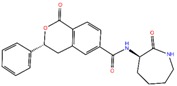 ZINC84525623	378.428	2	4	84.5	0	3

^a^ XP stands for Extra Precision. ^b^ HBD stands for Hydrogen Bond Donor. ^c^ HBA stands for Hydrogen Bond Acceptor. ^d^ Tpsa stands for the total polar surface area. ^e^ RB stands for Number of Rotatable bonds.

**Table 3:** The ADME/T properties of the top 5% selected compounds by ^a^ XP (Extra Precision) docking scores.

ZINC Id	QPpolrz (13 to 70)	QPlogS (−6 to 0.5)	QPlogPC16 (4 to 18)	QPlogPoct (8 to 43)	QPlogPw (5 to 48)	QPlogPo/w (−2 to 6)	QPlogKp (−8 to −1)	QPlog Khsa (−1.5 to 1.2)
ZINC10936382	43.299	−6.736	11.702	18.741	9.631	4.843	−1.346	0.813
ZINC30479078	48.327	−5.251	12.043	20.834	10.492	2.723	−2.811	0.636
ZINC41493045	40.603	−4.524	11.974	18.573	11.572	2.070	−2.946	−0.429
ZINC7424911	41.415	−5.717	12.778	19.790	12.387	3.337	−2.263	0.450
ZINC84525623	42.420	−3.863	12.527	21.290	15.816	2.284	−3.107	−0.003

ADME/T stands for Adsorption, Distribution, Metabolism, Excretion, and Toxicity. Lower and upper limits of the corresponding descriptor is given in the parentheses. ^a^ XP stands for Extra Precision docking.

**Table 4:** The molecular interaction between NDM-1 and different inhibitors from the ZINC database.

Compounds/ZINC IDs	Molecular Interactions	Nature of Interactions	Distance (Å)	Docking Binding Energy (∆*G*), kcal/mol	Docking Binding Affinity (*K*_d_), M^−1^	MM-GBSA Binding Energy, kcal/mol
Control (Hydrolyzed Meropenem)	Lys211:HZ1–Lig:O Asn220:HN–Lig:O Asn220:HD21–Lig:O Lig:H–His250:NE2 Lig:H–His120:NE2 Lig:H–His189:NE2 ZN2–Lig:O Trp93–Lig:C	Hydrogen Bond Hydrogen Bond Hydrogen Bond Hydrogen Bond Hydrogen Bond Hydrogen Bond Other (Metal–Acceptor) Hydrophobic (Pi–Alkyl)	2.72 1.99 1.85 2.59 2.76 2.32 2.92 5.25	−6.413	5.05 × 10^4^	−52.971
ZINC10936382	Gln123:HN–Lig:O Asn220:HD22–Lig:O ZN2–Lig:O ZN1–Lig His122–Lig His250–Lig Val73–Lig His122–Lig Lig–Val73	Hydrogen Bond Hydrogen Bond Other (Metal–Acceptor) Electrostatic Hydrophobic (Pi–Pi Stacked) Hydrophobic (Pi–Pi Stacked) Hydrophobic (Alkyl) Hydrophobic (Pi–Alkyl) Hydrophobic (Pi–Alkyl)	2.08 2.93 2.86 4.97 4.94 4.80 3.98 4.26 5.18	−8.322	1.27 × 10^6^	−61.432
ZINC30479078	Gln123:HN–Lig:O Asp124:HN–Lig:O Asp124:OD2–Lig Val73:CG1–Lig His122–Lig Lig–Val73	Hydrogen Bond Hydrogen Bond Electrostatic Hydrophobic (Pi–Sigma) Hydrophobic (Pi–Alkyl) Hydrophobic (Pi–Alkyl)	2.67 2.18 3.70 3.61 4.04 5.00	−9.046	4.31 × 10^6^	−70.643
ZINC41493045	His122:HD1–Lig:O Asn220:HD21–Lig:O ZN1–Lig:O ZN2–Lig His250–Lig Lys211–Lig His250–Lig	Hydrogen Bond Hydrogen Bond Other (Metal–Acceptor) Electrostatic Hydrophobic (Pi–Pi Stacked) Hydrophobic (Alkyl) Hydrophobic (Pi–Alkyl)	2.66 1.82 3.08 3.76 4.42 5.26 5.15	−7.714	4.54 × 10^5^	−62.523
ZINC07424911	Lig:HN–Asp212:OD2 Lig:HN–His250:O Asn220:HN–Lig Asp124:OD2–Lig His250–Lig Trp93–Lig Trp93–Lig Ala215–Lig	Hydrogen Bond Hydrogen Bond Hydrogen Bond Electrostatic Hydrophobic (Pi–Pi Stacked) Hydrophobic (Pi–Pi T–shaped) Hydrophobic (Pi–Pi T–shaped) Hydrophobic (Alkyl)	2.76 2.72 2.93 4.24 3.97 5.47 4.84 4.34	−8.254	1.13 ×10^6^	−60.619
ZINC84525623	Gln123:HN–Lig:O Gln123:HN–Lig:O Asp124:HN–Lig:O Lys211:HZ1–Lig:O Asn220:HN–Lig:O His189:CE1–Lig:O ZN2–Lig Asp124:OD2–Lig Val73:CG1–Lig His250–Lig His250–Lig Lig–Val73 His250–Lig	Hydrogen Bond Hydrogen Bond Hydrogen Bond Hydrogen Bond Hydrogen Bond Carbon–Hydrogen Bond Electrostatic Electrostatic Hydrophobic (Pi–Sigma) Hydrophobic (Pi–Pi Stacked) Hydrophobic (Pi–Pi T-shaped) Hydrophobic (Alkyl) Hydrophobic (Pi–Alkyl)	2.66 2.16 2.08 2.71 2.10 3.57 3.66 4.33 3.58 5.47 5.26 5.46 4.72	−8.790	2.80 × 10^6^	−96.388

**Table 5:** The steady-state enzyme kinetics of NDM-1 in the presence and absence of inhibitor ZINC84525623.

Substrates	NDM-1			NDM-1 + ZINC84525623 *		
	*K*_m_ (µM)	*k*_cat_ (s^−1^)	*k*_cat_/*K*_m_ (µM^−1^ s^−1^)	*K*_m_ (µM)	*k*_cat_ (s^−1^)	*k*_cat_/*K*_m_ (µM^−1^ s^−1^)
Ampicillin Cefotaxime Imipenem Meropenem Nitrocefin	88.9 ± 3.1 57.7 ± 3.3 68.4 ± 4.6 58.4 ± 4.1 29.0 ± 2.0	438.2 ± 10.5 330.8 ± 16.4 665.8 ± 14.4 285.8 ± 17.3 279.7 ± 18.2	4.93 ± 0.21 5.73 ± 0.43 9.73 ± 0.69 4.89 ± 0.45 9.64 ± 0.91	111.3 ± 6.2 87.9 ± 5.7 93.6 ± 5.1 80.3 ± 6.4 54.9 ± 4.7	97.8 ± 6.1 104.5 ± 6.3 123.1 ± 7.8 125.7 ± 6.0 123.5 ± 6.4	0.88 ± 0.07 1.19 ± 0.10 1.32 ± 0.11 1.56 ± 0.14 2.25 ± 0.22

* ZINC84525623 inhibitor was used at a final concentration of 20 µM.

## Supplementary Materials

Supplementary materials can be found at http://www.mdpi.com/1422-0067/20/4/819/s1.
