# Supplementary material for: High-Throughput Virtual Screening, Molecular Dynamics Simulation, and Enzyme Kinetics Identified ZINC84525623 as a Potential Inhibitor of NDM-1
Source: Int J Mol Sci. 2019 Feb 14;20(4):819. doi: 10.3390/ijms20040819 (PMC6412273; doi:10.3390/ijms20040819)
Supplement: Supplementary file 1 [file ijms-20-00819-s001.pdf]

Article

High-Throughput Virtual Screening, Molecular Dynamics Simulation, and Enzyme Kinetics Identified ZINC84525623 as a Potential Inhibitor of NDM-1

Md Tabish Rehman [1,*], Mohamed F AlAjmi [1], Afzal Hussain [1], Gulam Mohmad Rather [2] and Meraj A Khan [3,*]

[1] Department of Pharmacognosy, College of Pharmacy, King Saud University, Riyadh 11451, Saudi Arabia; malajmii@ksu.edu.sa (M.F.A.); afihussain@ksu.edu.sa (A.H.);

[2] Rutgers Cancer Institute of New Jersey, Rutgers, The State University of New Jersey, New Brunswick, NJ 08901, USA; ratherbiotech@gmail.com

[3] Program in Translational Medicine, Peter Gilgan Centre for Research and Learning, The Hospital for Sick Children, Toronto, ON M5G 0A4, Canada

* Correspondence: mrehman@ksu.edu.sa or m.tabish.rehman@gmail.com (M.T.R.); meraj.khan@sickkids.ca (M.A.K.); Tel.: +966-556814200 (M.T.R.); +1-416-813-7654 ext.303388 (M.A.K.); Fax: +966-14677245 (M.T.R.); +1-416-813-5771 (M.A.K.)

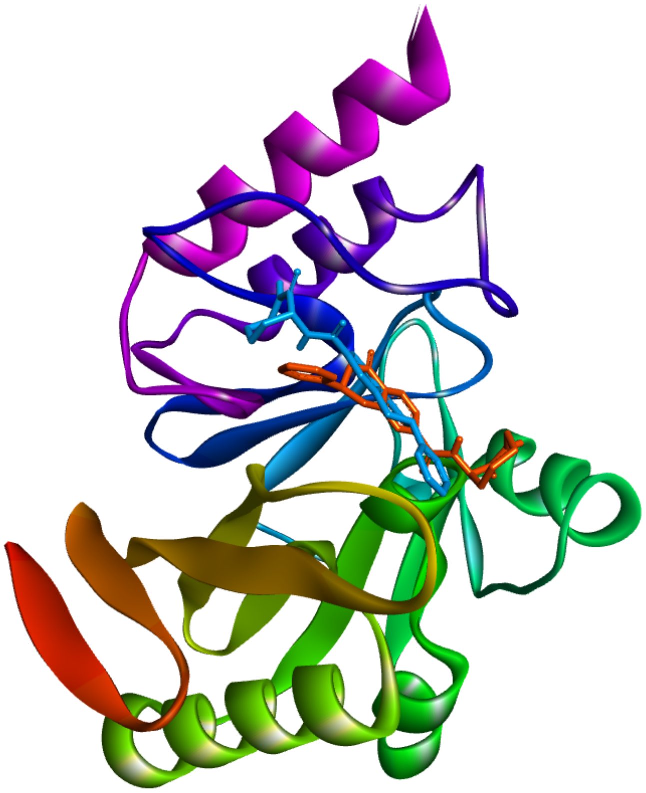

Figure S1: Superimposition of ZINC84525623 before (orange) and after (blue) molecular dynamics (MD) simulation.

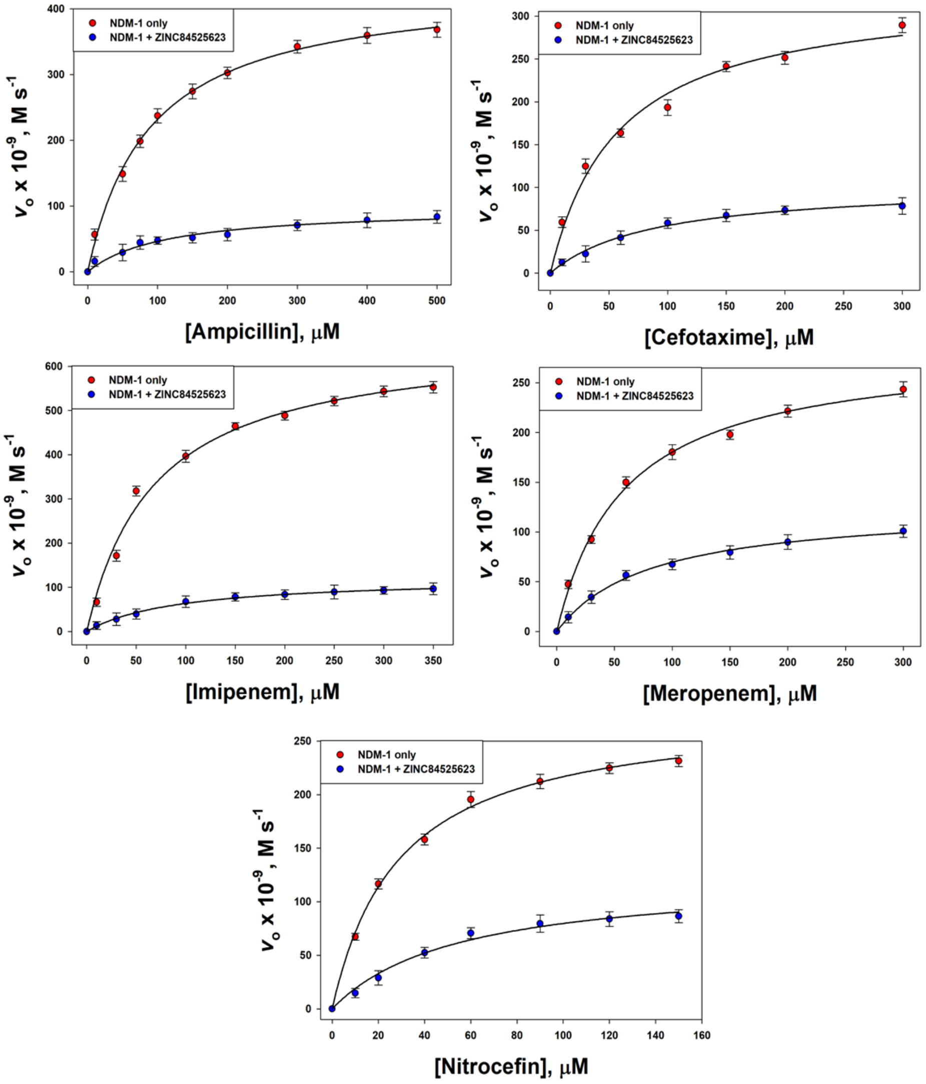

Figure S2: Steady-state enzyme kinetics of NDM-1 in the absence and presence of ZINC84525623.

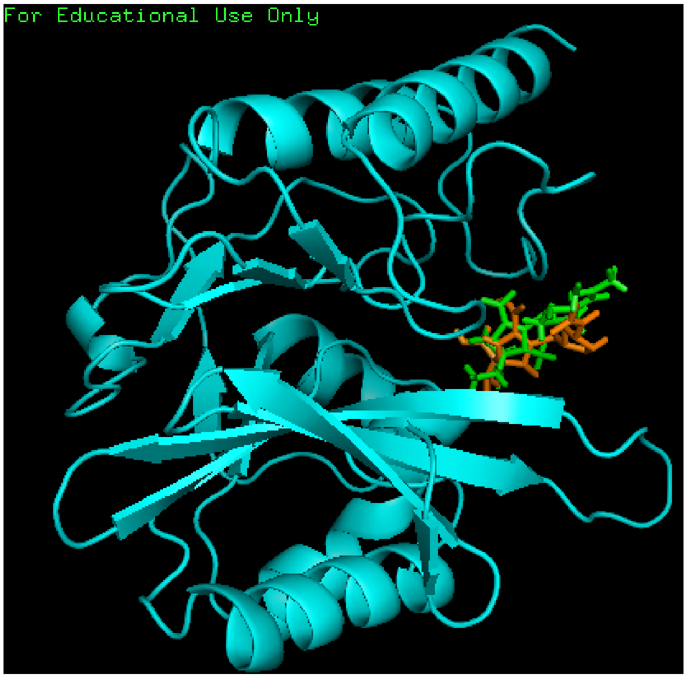

Figure S3: Validation of docking protocol by extracting and re-docking the bound inhibitor (i.e., Meropenem) at the active site of NDM-1 using XP docking. A comparison between crystal pose and docked pose of Meropenem shows that it occupied a similar position at the active site of NDM-1 in the docked conformations, as was present in the X-ray crystal structure.

Cloning Strategy:

NdeI—His tag—NDM-1_No signal—Stop codon—HindIII

vector: pET30a

DNA sequence: 762bp

CATATGCATCATCACCATCACCAC**GGTGAAATCCGTCCGACCATTGGTCAGCAGATGGAGACCGGTGATCAACGCTTTGGCG
ACCTGGTGTTCCGTCAACTGGCGCCGAATGTGTGGCAGCACACCAGCTACCTGGACATGCCGGGTTTCGGTGCGGTTGCGA
GCAACGGTCTGATTGTGCGTGACGGTGGCCGTGTTCTGGTGGTTGATACCGCGTGGACCGACGATCAGACCGCGCAAATCC
TGAACTGGATTAAGCAGGAGATCAACCTGCCGGTGGCGCTGGCGGTGGTTACCCACGCGCACCAAGACAAAATGGGTGGT
ATGGATGCGCTGCACGCGGCGGGTATCGCGACCTACGCGAACGCGCTGAGCAACCAGCTGGCGCCGCAAGAGGGTATGGT
GGCGGCGCAACACAGCCTGACCTTCGCGGCGAACGGTTGGGTGGAACCGGCGACCGCGCCGAACTTCGGTCCGCTGAAGG
TGTTCTACCCGGGTCCGGGCCACACCAGCGACAACATTACCGTTGGTATCGACGGCACCGATATTGCGTTTGGTGGCTGCCT
GATCAAAGATAGCAAGGCGAAAAGCCTGGGTAACCTGGGCGACGCGGATACCGAACACTATGCGGCGAGCGCGCGTGCGT
TCGGTGCGGCGTTTCCGAAGGCGAGCATGATTGTTATGAGCCACAGCGCGCCGGATAGCCGTGCGGCGATTACCCACACCG
CGCGTATGGCGGATAAACTGCGT**TAATGAAAGCTT

Protein details and sequence:

Protein Length=249   MW=26559.7   Predicted pI=6.68

**MHHHHHHGEIRPTIGQQMETGDQRFGDLVFRQLAPNVWQHTSYLDMPGFGAVASNGLIVRDGGRVLVVDTAWTDDQTAQI
LNWIKQEINLPVALAVVTHAHQDKMGGMDALHAAGIATYANALSNQLAPQEGMVAAQHSLTFAANGWVEPATAPNFGPLK
VFYPGPGHTSDNITVGIDGTDIAFGGCLIKDSKAKSLGNLGDADTEHYAASARAFGAAFPKASMIVMSHSAPDSRAAITHTARM
ADKLR..**

Figure S4: The cloning strategy adopted by GenScript (USA) to clone, express, and purify NDM-1. The given sequence of NDM-1 gene was used for cloning purpose. The sequence and other details of the expressed NDM-1 protein is also shown.

SDS-PAGE & Western blot Analysis:

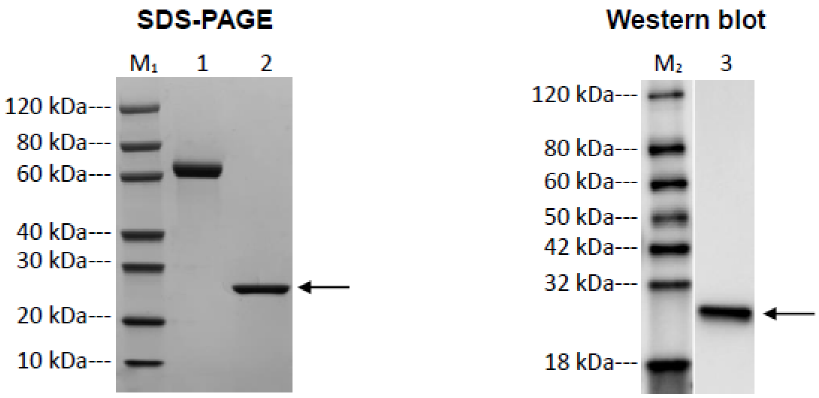

Fig.1 SDS-PAGE and Western blot analysis of NDM-1_No signal

Lane M_1: Protein Marker, GenScript, Cat. No. M00516

Lane M_2: Protein Marker, GenScript, Cat. No. M00521

Lane 1: BSA (2.00 µg)

Lane 2: NDM-1_No signal (Reducing conditions, 2.00 µg)

Lane 3: NDM-1_No signal (Reducing conditions)

Primary antibody: Mouse-anti-His mAb (GenScript, Cat.No. A00186)

Figure S5: SDS-PAGE and Western blot analysis of the expressed NDM-1 protein, confirming its size and nature.

Table 1: XP docking parameters of shortlisted compounds after HTVS and SP docking.

S. No.	ZINC ID	Docking score*	Glide g-score*	Glide e-model*	XP g-Score*
1.	10056647	-6.744	-6.744	-60.067	-6.744
2.	10298772	-6.962	-6.962	-71.278	-6.962
**3.**	**10936382**	**-8.322**	**-8.322**	**-68.183**	**-8.322**
4.	11820118	-6.961	-7.280	-61.139	-7.280
5.	12406851	-6.410	-6.410	-59.446	-6.410
6.	12535179	-6.230	-6.641	-69.803	-6.641
7.	12590790	-5.692	-5.692	-62.235	-5.692
8.	13008841	-5.180	-5.180	-59.592	-5.180
9.	13363540	-5.203	-5.203	-63.387	-5.203
10.	13700245	-4.980	-4.980	-55.097	-4.980
11.	14621235	-4.814	-4.843	-59.923	-4.843
12.	14968695	-5.852	-5.852	-73.108	-5.852
13.	15546068	-5.573	-5.573	-57.753	-5.573
14.	16014694	-6.068	-6.068	-56.701	-6.068
15.	16968888	-5.869	-5.869	-57.466	-5.869
16.	16969550	-6.020	-6.020	-54.122	-6.020
17.	17859734	-6.443	-6.443	-71.251	-6.443
18.	19293272	-5.772	-5.814	-54.657	-5.814
19.	20719470	-5.160	-5.160	-68.670	-5.160
20.	21884979	-5.657	-5.657	-57.850	-5.657
21.	21885339	-6.426	-6.426	-61.384	-6.426
22.	2310269	-6.403	-6.403	-57.505	-6.403
23.	23143203	-5.649	-5.651	-53.858	-5.651
24.	24756392	-5.234	-5.550	-52.198	-5.550
25.	27589567	-4.849	-4.850	-59.910	-4.850
26.	**30479078**	**-9.046**	**-9.046**	**-66.578**	**-9.046**
27.	31587919	-6.131	-6.131	-61.297	-6.131
28.	32830532	-5.999	-5.999	-59.226	-5.999
29.	32830535	-5.999	-5.999	-59.226	-5.999
30.	32925747	-6.360	-6.389	-62.000	-6.389
31.	32975301	-4.305	-4.305	-54.688	-4.305
32.	32996624	-3.612	-3.612	-49.223	-3.612
33.	33008314	-6.119	-6.119	-62.114	-6.119
34.	33311487	-7.136	-7.136	-58.803	-7.136
35.	3401702	-7.104	-7.104	-58.671	-7.104

36.	35479165	-3.321	-3.321	-52.917	-3.321
37.	35503898	-4.433	-4.433	-47.164	-4.433
38.	35503902	-4.333	-4.333	-49.678	-4.333
39.	38492621	-5.950	-5.950	-63.061	-5.950
40.	38498146	-5.333	-5.333	-62.719	-5.333
41.	38573638	-6.746	-6.746	-56.373	-6.746
42.	38652085	-4.971	-4.971	-51.495	-4.971
43.	38671609	-5.431	-5.475	-57.961	-5.475
44.	38708676	-4.612	-4.612	-55.988	-4.612
45.	38787768	-6.383	-6.383	-66.581	-6.383
46.	39876668	-5.813	-5.814	-58.387	-5.814
**47.**	**41493045**	**-7.714**	**-7.714**	**-64.597**	**-7.714**
48.	43014303	-5.720	-5.720	-58.917	-5.720
49.	43015264	-5.830	-5.830	-57.429	-5.830
50.	43731718	-5.555	-5.555	-49.168	-5.555
51.	44014285	-6.251	-6.251	-65.093	-6.251
52.	45408555	-5.758	-5.758	-59.964	-5.758
53.	47482030	-6.933	-6.933	-56.222	-6.933
54.	48097483	-6.486	-6.486	-62.157	-6.486
55.	48356067	-7.029	-7.029	-69.711	-7.029
56.	49006330	-5.088	-5.088	-51.610	-5.088
57.	49481379	-6.347	-6.347	-62.587	-6.347
58.	49509500	-6.151	-6.151	-71.743	-6.151
59.	62901101	-6.802	-6.802	-64.234	-6.802
60.	64390886	-6.976	-6.976	-57.361	-6.976
61.	6492299	-5.564	-5.623	-50.884	-5.623
62.	64990456	-5.889	-5.889	-69.662	-5.889
63.	64998230	-5.802	-5.802	-66.231	-5.802
64.	65060706	-5.920	-5.920	-61.930	-5.920
65.	65114888	-6.307	-6.331	-40.701	-6.331
66.	65235659	-5.628	-5.629	-54.039	-5.629
67.	66507291	-6.478	-6.478	-60.422	-6.478
68.	66544764	-5.420	-5.420	-54.366	-5.420
69.	66605279	-7.280	-7.280	-70.096	-7.280
70.	67281340	-6.197	-6.197	-56.899	-6.197
71.	68128815	-5.614	-5.614	-63.837	-5.614
72.	68168251	-7.041	-7.041	-66.658	-7.041
73.	68483776	-6.988	-6.988	-52.126	-6.988
74.	68483889	-6.035	-6.036	-59.120	-6.036
75.	68532966	-6.708	-6.709	-63.559	-6.709

76.	68781030	-5.186	-5.186	-56.069	-5.186
77.	72349207	-6.218	-6.233	-55.223	-6.233
78.	72356469	-6.691	-7.020	-61.262	-7.020
79.	72356480	-6.674	-6.674	-65.278	-6.674
80.	72356485	-6.672	-6.675	-64.542	-6.675
81.	72395317	-6.736	-6.736	-64.262	-6.736
82.	73211718	-5.329	-5.340	-64.576	-5.340
**83.**	**7424911**	**-8.254**	**-8.265**	**-63.254**	**-8.254**
84.	74375116	-6.415	-6.415	-56.727	-6.415
85.	75492474	-7.044	-7.068	-62.055	-7.068
86.	75595463	-3.668	-3.690	-45.571	-3.690
87.	7717719	-7.006	-7.203	-62.389	-7.203
88.	77865922	-5.198	-5.198	-55.932	-5.198
89.	84512487	-5.734	-5.734	-54.844	-5.734
**90.**	**84525623**	**-8.790**	**-8.790**	**-64.740**	**-8.790**
91.	8745450	-4.786	-4.786	-49.146	-4.786
92.	89878855	-6.545	-6.574	-64.425	-6.574
93.	90096655	-5.093	-5.093	-49.896	-5.093
94.	90100211	-5.417	-5.417	-52.292	-5.417
95.	90100480	-3.620	-3.620	-46.004	-3.620
96.	90369391	-5.524	-5.524	-62.230	-5.524
97.	91673029	-7.085	-7.085	-57.240	-7.085
98.	93924915	-6.403	-6.403	-60.190	-6.403
99.	9563026	-5.878	-5.878	-62.762	-5.878
100.	77969431	-6.564	-6.564	-61.802	-6.564
**101.**	**Control (Meropenem)**	**-6.413**	**-6.413**	**-56.140**	**-6.413**

*All the energies are in kcal/mol. The compounds shown in bold were shortlisted for further analysis. HTVS, SP, and XP stands for High Throughput Virtual Screening, Standard Precision, and Extra Precision respectively.
